# The perspectives of children and young people affected by parental life-limiting illness: An integrative review and thematic synthesis

**DOI:** 10.1177/0269216320967590

**Published:** 2020-11-19

**Authors:** Steve Marshall, Rachel Fearnley, Katherine Bristowe, Richard Harding

**Affiliations:** Florence Nightingale Faculty of Nursing, Midwifery & Palliative Care, Cicely Saunders Institute of Palliative Care, Policy & Rehabilitation, King’s College London, London, UK

**Keywords:** Child, adolescent, family, parents, bereavement, critical illness, review

## Abstract

**Background:**

Although the death of a parent during childhood is relatively commonplace, the voices of children affected by parental life-limiting illness are under-represented in research evidence. Guidance for healthcare professionals is largely based upon professional opinion rather than the experience of children themselves.

**Aim:**

To synthesise and appraise the literature from primary research with children about their experience of having a parent with a life-limiting illness.

**Design:**

Integrative review and thematic synthesis. Registered on PROSPERO (CRD42019094581).

**Data sources:**

PsychINFO, Medline, Embase, Scopus and Web of Science were searched, supplemented by searches of grey literature and systematic reviews. There were no restrictions on publication date, and study quality was appraised using the Hawker checklist. Studies reporting the findings of primary research with participants under 18, whose parent has a life-limiting illness, were eligible for inclusion.

**Results:**

Twenty-one papers met the inclusion criteria (*n* = 13 qualitative; *n* = 8 quantitative), reporting on *n* = 18 studies from high-income countries. Findings reveal that throughout parental life-limiting illness, children strive for agency, but are often shielded and excluded by adults. The experience of living with a dying parent is emotionally demanding for children and involves significant caregiving responsibilities. However these children are not passive, developing strategies to cope with the situation and wanting to be involved.

**Conclusions:**

The review has enabled the voices of children affected by parental life-limiting illness to be heard and will inform the development of guidance for parents and professionals.

**What is already known about the topic?**

- The experience of the death of a parent during childhood places adults at higher risk of poor outcomes, such as anxiety and depression.
- The period prior to a parent’s death may be of particular significance and can impact on how children cope in bereavement.
- Many healthcare professionals consider themselves unqualified to provide guidance to dying parents around supporting their children.

**What this paper adds?**

- Children living with parental life-limiting illness desire open communication and want to be more involved.
- Children living with parental life-limiting illness display their agency and develop strategies to manage a difficult situation.
- There is a tension between children’s desire for agency and the parental desire to shield and protect children from the illness.

**Implications for practice, theory or policy**

- Under international resolutions, children have a fundamental right to be involved in matters affecting them, but this may not be recognised when a parent is dying.
- Children maintaining agency, despite parental life-limiting illness, is a useful conceptualisation of their experience and could influence a change in practice.
- Recognising the caring role that children undertake when a parent is dying may lead to a more inclusive approach.

## Background

Over 40,000 children and young people under the age of 18 (referred to as children hereafter) in the United Kingdom (UK) experience parental death annually, and the equivalent of one child in every UK classroom has been bereaved of a parent.^1^ The prevalence of parental death as a child is similar in other high-income countries such as the USA,^2^ but is higher in low and middle-income countries, and nations in sub-Saharan Africa with a high incidence of HIV.^3^ The death of a parent during childhood can have a profound and enduring impact, and is often associated with anxiety, depression, self-harm, anti-social behaviour and below-average educational attainment.^2,4–9^ Poor outcomes in people experiencing a significant bereavement in childhood have been attributed to issues such as inconsistent care and attention, separation from family, disruption in education, a lack of communication and information about the illness, uncertainty around the prognosis and a lack of preparation for the parental death.^10,11^

The period prior to the death of a parent is particularly significant. Living with a seriously ill parent may change the roles expected of children and result in burdensome caring responsibilities.^12^ The pressure and stress associated with being a young carer can impact upon a child’s health and wellbeing,^13^ but unfortunately young carers can go unnoticed and their needs are often not addressed.^14,15^ How children are supported at the end of a parent’s life may have a direct influence on how they cope after the bereavement.^16,17^ Being unaware and unprepared for a parent’s imminent death may impact upon children’s psychosocial wellbeing in bereavement^18,19^ and interventions aimed at improving family communication may be beneficial.^20^ However, parents often find this period particularly difficult and are unsure how best to support their children.^21,22^ Parents can be overwhelmed by the situation, struggling with their own emotions and receiving conflicting advice from friends and family.^23^ Professional advice and support can be lacking, as healthcare professionals often consider themselves inexperienced and unqualified to provide guidance in supporting these children.^24,25^ Even experienced palliative care professionals can find it difficult to provide holistic family-centred care when children are involved and are often unsure what advice to give to parents.^26,27^ The result is that the needs of children may not be appropriately addressed as their parent is dying.

Despite the large number of children experiencing parental death each year and the potential long-term negative impact on them, little primary research has been conducted with this population. The voices of children facing the death of a parent are under-represented in prior research studies.^28^ It is rare for children to be asked about their experience when a parent has a life-limiting illness, and most primary research in this area occurs after the death and when the bereaved child has reached adulthood. Due to the paucity of primary research, any recommendations and guidelines for healthcare professionals have been based upon professional opinion rather than evidence informed by the experiences of children themselves.^3^ Prior to conducting any further research with this cohort, it is necessary to establish what data has been collected previously and develop an understanding of the findings.

The aim of this review is therefore to synthesise and appraise the literature from primary research with children about their experience of having a parent with a life-limiting illness. The findings will be used to identify gaps in the evidence to guide future research and to inform the development of guidance for parents and professionals.

## Methods

The review followed the PRISMA-P Guidelines^29^ and the protocol was registered on the International Prospective Register of Systematic Reviews (PROSPERO) (https://www.crd.york.ac.uk/prospero/display_record.php?ID=CRD42019094581) in January 2019. An integrative review method^30^ was used, as this broad review methodology enables the inclusion of findings from studies reporting diverse methodologies.

### Search strategy

A search of electronic databases was conducted on 31 January 2020. The search strategy was developed with an information specialist using medical subject headings (MeSH) and text words relating to children and adolescents facing the death of a parent. Reviews on related subjects were consulted to help improve the search.^24,25^ The search strings have been provided as a supplementary file. The following electronic bibliographic databases were searched: PsychINFO, Medline, Embase and Scopus. The citation database Web of Science was also searched, as well as a search of grey literature (www.opengrey.eu). The search of electronic databases was supplemented by searching PROSPERO, the Cochrane Database of Systematic Reviews and Epistemonikos (www.epistemonikos.org) for on-going or recently completed systematic reviews. To ensure no key references were missed, the reference lists of included studies and relevant reviews were hand searched. Any articles not identified by the search, but already known to the research team, were also included.

### Assessment

The inclusion and exclusion criteria applied to the search are detailed in Table 1. Two authors (SM and RF) independently screened the titles and abstracts. Full text of the remaining articles was appraised against the inclusion criteria (SM and RF). The studies to be retained were agreed through a process of discussion and consensus (SM, KB, RH and RF). A life-limiting illness was defined as a medical condition for which there is no cure and from which a person is expected to die prematurely, to differentiate from papers focussed upon survivorship and long-term/chronic illness. The methodological rigour of included studies was independently assessed (SM and RF) using the checklist devised by Hawker et al.^31^ Reporting assessments were conducted independently using the Consolidated Criteria for Reporting Qualitative Research (COREQ) checklist^32^ for qualitative studies (SM and RF) and the Strengthening the Reporting of Observational Studies in Epidemiology (STROBE) statement^33^ for quantitative studies (RH and RF). The scores for each included study are included in Table 2, enabling the reader to assess and compare studies. Due to the small numbers of studies reporting primary data from children, articles were not excluded on the basis of the assessment of rigour or reporting. However the quality of studies was factored into the analysis. The senior author (RH) adjudicated decisions on inclusion, exclusion, reporting and quality rating.

**Table 1.** Inclusion and exclusion criteria.

Inclusion	Exclusion
Study designs:Studies reporting the findings of primary research, including quantitative, qualitative and mixed methods research designsParticipants:Participants who are under the age of 18 at the time of the study and who have a parent or primary carer with a life-limiting illness [defined as a medical condition for which there is no cure and from which a person is expected to die prematurely]Publication status:All published studies reporting primary data and unpublished materials (grey literature) reporting primary data	Study designs:Systematic reviewsCase studiesParticipants:Conditions where the parent or primary carer is not expected to diePublication type:Commentaries, letters, editorials and professional opinion

**Table 2.** Characteristics of included studies (*n* = 21).

StudyAuthorsCountryQuality (Hawker Scale 9–36)Reporting assessment(COREQ 32-item checklist for qualitative studies;STROBE 22-item checklist for quantitative studies)	Aims and objectives	ParticipantsNumber of participantsAge range of participantsGender of participantsEthnicity of participantsParental/primary carer disease	Methods	Relevant findings	Strengths and limitations
Bauman et al.^34^USA and ZimbabweHawker score: 24/36STROBE score: 9.5/22	To document the caring responsibilities and psychological status of children of mothers living with HIV/AIDS	*n* = 100 participants(50 in Mutare, Zimbabwe, 50 in New York, USA)Ages 8–1664% female, 32% male (NY)58% female, 42% male (Mutare)61% Black, 33% Hispanic (NY)96% Zimbabwean, 4% Mozambican (Mutare)Mothers with HIV/AIDS	Quantitative:unvalidated questionnaire designed for the study about caring responsibilities, along with the Children’s Depression Inventory, the Inventory of Parent and Peer Attachment, the Parentification Scale and the Emotional Parentification Questionnaire	Participants provided personal care, performed household tasks and acted as confidants. The majority reported that they had too much responsibility, impacting upon after-school and peer activities. Participants felt more capable because of their responsibilities. Depression rates were high in all participants, but two-thirds of Mutare children had clinically significant depression scores	Large, ethnically diverse sample. International design enables cross-cultural comparison and ethnic diversity. Findings section provides detailed analysis. Limitations in quality of reporting
Beale et al.^35^USAHawker score: 21/36COREQ score: 11/32	To report the experiences of children of parents with terminal cancer	*n* = 28 participantsAges 3–1815 male, 13 femaleNo information about ethnicity of participantsParents with advanced cancer	Qualitative:semi-structured interviews	23/28 participants sought reassurance. 22/28 participants considered themselves to be caregivers. High levels of aggressive behaviour and separation anxiety were displayed. Participants wanted to protect and help their dying parent. All had some understanding of their parent’s condition and were frightened by the outcome	Limited information from the study. Sample restricted to children referred for therapeutic support at one site and limited to parental cancer.Weak, poorly reported methodology.No information about participant ethnicity
Buchwald et al.^36^DenmarkHawker score: 28/36COREQ score: 17.5/32	To describe and understand how children handle life when a parent is dying	*n* = 7 participantsAges 11–174 male, 3 femaleNo information about ethnicity of participantsNo information about parental disease	Qualitative:semi-structured interviews and video diaries	When a parent is dying, the family home can become ‘death’s waiting room’. Not discussing the imminent death with children leaves them alone with their thoughts and feeling unprepared.	No information on how the small sample was selected. Some contradictions in the findings. Use of video diaries is an innovative and child-friendly method. No information on ethnicity or parental disease
Calvo et al.^37^ItalyHawker score: 27/36STROBE score: 14.5/22	To investigate the psychological impact of having a parent with ALS on school-age children and adolescents	*n* = 23 participantsAges 5–1717 male, 6 femaleParticipants were all whiteParents with ALS	Quantitative: the Youth Self Report questionnaire and a personality assessment using the Rorschach test	Participants displayed clinically significant emotional and behavioural problems, including internalizing problems, anxiety, withdrawal and depressive symptoms	Small sample size limited to 12 families from one site. Findings well presented. All white sample
Chowns^38^UKHawker score: 20/36COREQ score: 14/32	To capture the lived experience of children with a seriously ill parent	*n* = 9 participantsAges 7–15No information about gender or ethnicity of participantsSeriously ill parents	Qualitative:action research	Participants struggled with the sense of isolation and the uncertainty of parental life-limiting illness. Their preference was for support and understanding rather than protection. Participants wanted to be informed and involved as soon as possible	Small sample size from one site. Weak methodology and unconventional approach to reporting. No information about gender, ethnicity or parental disease. Findings supported by appropriate quotes from participants
Christ et al.^39^USAHawker score: 21/36COREQ score:15.5/32	To explore the characteristic psychosocial reactions to a parent’s advanced illness	*n* = 120 participantsAges 11–17Gender not specified85% White, 7% Black, 7% Hispanic, 1% AsianParents with terminal cancer	Qualitative: semi-structured interviews	Participants had empathy for their parent’s suffering, but felt guilty about some emotions and behaviours. The adolescent need for independence conflicted with the emotional involvement with the ill parent and the increased caregiving responsibilities	Methodologically weak, limited information about the methods used. Large sample but limited to one site. Unconventional approach to reportingSample not ethnically diverse
Eklund et al.^40^SwedenHawker score: 27/36STROBE score: 17/22	To explore children’s reports of illness-related information and family communication when living with a parent with a life-threatening illness	*n* = 48 participantsAges 7–1921 female, 27 maleNo information about ethnicity of participantsParents with cancer and neurological, pulmonary, cardiovascular and gastrointestinal life-threatening illnesses	Quantitative: validated questionnaire designed for the study	The majority of participants had been told about the illness by a parent. Two thirds of the participants wanted to know more about the illness. A quarter of the children said that they had questions about the illness that they did not dare ask. Half of the younger children (8–12 years) felt unable to talk about how they felt or show their feelings to someone in the family. All the adolescents were able to talk to someone in the family, although three were not satisfied with these conversations	Large sample size, broad age range and diversity of life-threatening illnesses. Broad information provided in the results section. Good conclusion linked to clinical practice. Some discrepancies with the ages of childrenNo information included about participant ethnicity
Kavanaugh et al.^41^USAHawker score: 30/36COREQ score: 15/32	To explore the support needs of children and adolescents who provide care to a parent with Huntington’s disease	*n* = 40 participants9 male, 31 femaleEthnicity not specifiedAges 12–20Parents with Huntington’s disease	Qualitative: semi-structured interviews	Participants emphasized the need for assistance from others. Participants identified a need for information and advice about the illness and caregiving. Friends were not supportive and distanced themselves	Large sample size, from a wide geographical area. 77% of participants were female. No information about participant ethnicity. Good inclusion of quotes from participants. Findings well presented
Kennedy, and Lloyd-Williams^42^UKHawker score: 28/36COREQ score: 14.5/32	To explore how children cope with advanced cancer in a parent	*n* = 11 participantsAges 8–189 female, 2 male9 White, 1 Black Caribbean, I Latin AmericanParents with advanced cancer	Qualitative: semi-structured interviews	The news that their parent had cancer was very distressing for participants. Coping strategies included – reasoning, having a positive attitude, information, getting on with things, maintaining normality, distractions, talking about it or not talking about it, maximising time with the parent. Many participants wanted to talk to somebody outside the family but were unable to access supportMost believed that basic daily life continued as ‘normal’	Small sample size, mainly white and female participants. Some examples of children’s narratives did not align closely with the commentary
Kennedy and Lloyd-Williams^43^UKHawker score: 32/36COREQ score: 14.5/32	To explore children’s information needs when a parent is diagnosed with advanced cancer	*n* = 11 participantsAges, gender and ethnicity not specifiedParents with advanced cancer	Qualitative: semi-structured interviews	All participants wanted more information about the parental illness, so that they could be prepared for the future and feel included in the family. Participants wanted information about how to help their parents, the treatment, length of hospital stays, and their own health. Parents were considered the best source of information, followed by healthcare professionals. Communication with someone outside the family was identified as beneficial	Small sample size, limited information about characteristics of participants. Limited information about recruitment. Good use of quotes
Kühne et al.^44^GermanyHawker score: 25/36STROBE score: 16.5/22	To explore adolescent psychosocial adjustment and quality of life across the disease stages of parental cancer	*n* = 86 participantsAges 11–2156% female, 44% maleEthnicity not specifiedParents with non-curative cancer	Quantitative: the Strength and Difficulties Questionnaire, Kidscreen-10, Kidcope, the Hospital Anxiety and Depression Scale and the Family Assessment Device	Participants reported distress throughout the parental disease process. Participants reported significantly less psychosocial problems and better quality of life during the palliative disease stage	Large sample taken from multiple sites. All participants were receiving child-centred counsellingComposition of sample well presented, although ethnicity omittedResults clearly presented
Kühne et al.^45^GermanyHawker score: 25/36STROBE score: 13.5/22	To examine family functioning and family members’ perspectives across the parental cancer disease stages	*n* = 31 participantsAges 11–1813 female, 18 maleEthnicity not specifiedParents in palliation	Quantitative: the Family Assessment Device	Disease stage was not a significant predictor of family functioning and relationships can be strengthened during palliation. Participants reported that incidents because of the disease tend to become more dominant, and spending time with the family became more important. Parental palliative disease is perceived by participants as both negative and positive with regards to family functioning	Sample section presents a clear account of the inclusion criteria but nothing about the composition of the sample. No information included about participant ethnicity. Examples of children’s narratives presented but these are limited
Phillips^46^USAHawker score: 28/36COREQ score: 21.5 /32	To gain an understanding of the experiences of adolescents with a parent with advanced cancer	*n* = 10 participantsAges 14–177 male, 3 femaleEthnicity not specifiedParents with advanced or metastatic cancer	Qualitative: semi-structured interviews	Four themes were identified by participants: cancer becomes the new normal and interrupts the life trajectory; the importance of contributing to the family’s well being; developing coping strategies to deal with emotions; and the cancer experience can have positive aspects	Small sample sizeOnly older adolescents included, with a larger number of malesRecruited through one organisation that was already providing support. Bias and ethics not addressed. No information included about participant ethnicity
Phillip and Lewis^47^USAHawker score: 25/36COREQ score: 17.5/32	To obtain the adolescent perspective on the impact of a parent’s advanced cancer on their life	*n* = 7 participantsAges 11–155 female, 2 maleEthnicity not specifiedParents diagnosed with Stage IV cancer	Qualitative: semi-structured interviews	Five major domains were identified by participants:parental cancer can be an emotional burden; cancer has a huge impact on everyone close to the parent; it is important to confront the cancer but also to periodically get away from it; talking about the cancer is difficult but necessary; and cancer can have positive aspects	Small sample comprised of early adolescents from white, middle class families with married parents. Predominantly female sample recruited through one out-patient clinicBias and ethics not addressed. No information included about participant ethnicity
Rainville et al.^48^CanadaHawker score: 25/36STROBE score: 16.5/22	To evaluate the impact of advanced parental cancer on adolescents’ psychological status	*n* = 28 participantsAges 12–1818 female, 10 maleEthnicity not specifiedParents with advanced cancer	Quantitative: the Indice de detresse psychologique de Sante ´ Quebec	Participants experienced higher psychological distress than the general population, especially if they are older (15–18). This may be due to a shift in roles in older adolescents, requiring them to take on more caring and household responsibilities. This may conflict with the desire for independence in this age group and cause increased psychological distress	Small sample size, specific to urban Quebec. Majority of participants were female. No information about ethnicityFunctional status of parent not accounted for. Only adolescents fully informed of parental cancer included
Sheehan and Draucker^49^USAHawker score: 33/36COREQ score: 15/32	To identify strategies to prepare children for life after parental death	*n* = 10 participantsAges 12–186 male, 4 femaleThe majority of participants were whiteParents with advanced cancer	Qualitative: semi-structured interviews	Time was important to participants, especially not having enough time with dying parent and making the most of the limited time. This was managed in four stages: coming to know time together is limited; spending more time together; extending time together; and giving up time together to end the suffering	Small sample size. Recruitment limited to adolescent participants at one hospice. Methods section is clear and concise, but lacking in reflexivity about the researchers. Findings are well presented but only one quotation included
Sheehan et al.^50^USAHawker score: 34/36COREQ score: 15.5/32	To explore how adolescents are told that their parent is dying	*n* = 26 participantsAges 12–1816 female, 10 male20 White, 4 Black, 1 Mixed Race, 1 not specifiedParents known to hospice	Qualitative: semi-structured interviews	Participants appear to know more about the poor prognosis than their parents are aware. Families have particular styles of disclosing the imminence of death and participants appear to adopt and mirror these styles	Recruitment limited to adolescent participants from one hospice. Methods section is clear and concise, but no information about the interviewer. Findings are well presentedSample not ethnically diverse
Sheehan et al.^51^USAHawker score: 34/36COREQ score: 15.5	To develop an explanatory model of coping strategies used by adolescents when a parent is dying	*n* = 30 participantsAges 12–1818 female, 12 male22 White, 5 Black, 2 Mixed Race, 1 not specifiedParents known to hospice	Qualitative: semi-structured interviews	Participants cope with parental illness by managing a ‘Well world’ and an ‘Ill world’. There are 5 stages in managing the two worlds: keeping the two worlds separate; ill world intrudes into well world; moving between the two worlds; being immersed in the ill world; and returning to the well world having been changed	Large sample.Recruitment of participants was limited to adolescents at one hospice. Good quotes from participants supporting the discussionSample not ethnically diverse
Siegel et al.^52^USAHawker score: 24/36STROBE score: 14/22	To compare the psychosocial adjustment of children with a terminally ill parent with a community sample	*n* = 62 participantsAges 7–1634 female, 28 maleAll participants were whiteParents with advanced cancer	Quantitative: the Children’s Depression Inventory, the State-Trait Anxiety Inventory and the Self-Esteem Inventory-Short Form	Self-reported levels of depressive symptomatology and anxiety were significantly higher in children with terminally ill parents than the control group	Only two parent families were included in the sample. Results section is brief, but discussion provides a comprehensive analysis. Sample not ethnically diverse
Siegel et al.^53^USAHawker score 26/36STROBE score: 15/22	To compare pre- and post-death levels of depression and anxiety in children experiencing parental cancer with a community sample	*n* = 97 participantsAges 7–1755 female, 42 maleEthnicity not specifiedParents with advanced cancer	Quantitative: the Children’s Depression Inventory and the State-Trait Anxiety Inventory	The terminal phase may be of greater psychological vulnerability for children than the period following the death. Children are particularly distressed by the physical changes that may occur during the terminal phase. Children may have high psychological vulnerability for longer, as medical interventions prolong the terminal phase	Large sample size, but limited to families receiving an intervention programme from one site. No information included about participant ethnicity
Turner^54^UKHawker score: 28/36COREQ score: 9.5/32	To interrogate young people’s perspectives on open communication within families when a parent is dying	*n* = 10 participantsAges 13–213 female, 7 maleEthnicity not specified(9 participants born in UK, one born in Indian sub-continent)9 parents with advanced cancer, one with MND	Qualitative: semi-structured interviews	Participants valued open communication, but this was not always regarded as positive. Participants were active agents, choosing to find out about their parent’s illness on their own terms. Participants preferred to exercise control over their knowledge of the illness and prognosis	Small sample sizeStudy aims not clearly stated. Good inclusion of quotes from participants. Methodological considerations are weak. No information included about participant ethnicity

### Data extraction, synthesis and analysis

**Figure 1.**
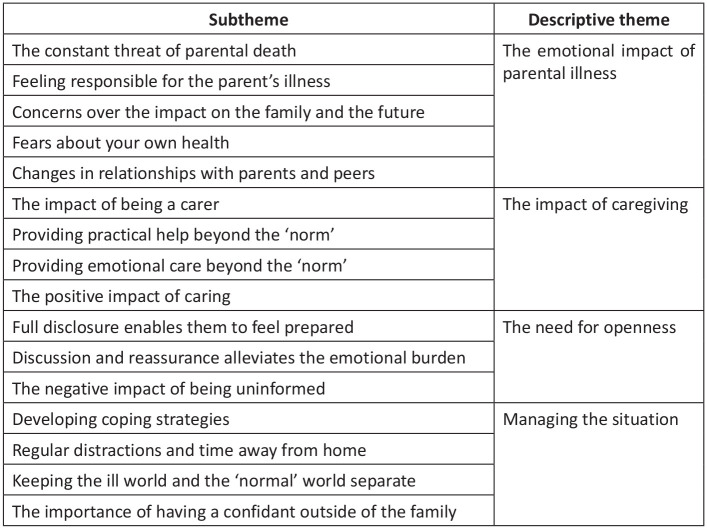
Descriptive subthemes and themes.

The data analysis stage was informed by integrative review methodology.^30^ Firstly, findings from quantitative studies were summarized descriptively. By comparing these descriptive data item by item, similar data were categorized together and broad categories were developed (SM and RF). Secondly, data (results and discussion sections) were extracted from the qualitative studies and imported into a common extraction table. The extracted data were independently coded line-by-line using *Microsoft Word*. By exploring patterns in the data, the codes were independently grouped into broad ‘candidate’ themes, and sub-themes were independently created under each candidate theme. In the third stage, the quantitative and qualitative data were integrated into a single framework (SM and RF). A process of data checking and investigator triangulation was conducted to ensure the framework adequately represented all themes from the quantitative and qualitative analysis (SM and RF). Once satisfied that the themes encompassed the data as a whole, they were finalized into descriptive themes (SM and RF). Any discrepancies were debated (SM and RF), with the senior author (RH) adjudicating and having final decision. The subthemes and their grouping into the final descriptive themes are illustrated in Figure 1. The final stage was to move beyond description of the data to a higher level of abstraction. Through an iterative process of review of descriptive themes, common patterns, relationships and processes by the project team, an integrated synthesis was achieved (SM, RF, KB and RH).

## Results

### Characteristics of retained studies

**Figure 2.**
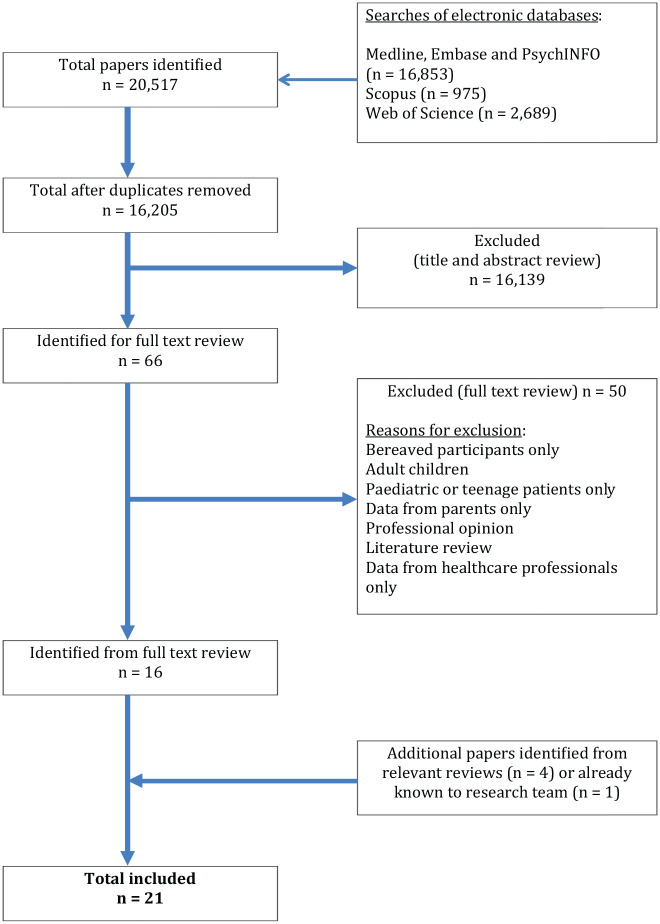
PRISMA flow diagram.

The PRISMA flowchart is illustrated in Figure 2. The search of the electronic databases identified *n* = 20,517 citations. After removal of duplicates and screening of titles and abstracts, *n* = 66 were subjected to full text review and *n* = 16 of these were identified as meeting the inclusion criteria. With *n* = 5 additional papers identified from relevant reviews and papers already known to the research team, a total of *n* = 21 publications met the inclusion criteria and were retained in the review.^34–54^ Of these, *n* = 13 are qualitative designs^35,36,38,39,41–43,46,47,49–51,54^ and *n* = 8 quantitative.^34,37,40,44,45,48,52,53^ These *n* = 21 publications report *n* = 18 studies. The studies originate from the USA (*n* = 9),^35,39,41,46,47,49–53^ UK (*n* = 3)^38,42,43,54^ and one each from Germany,^44,45^ Denmark,^36^ Canada,^48^ Sweden^40^ and Italy.^37^ One is a joint Zimbabwe/USA study.^34^ Parental disease is not always reported, but cancer is most prevalent with HIV, ALS, Huntington’s Disease and MND also represented. Where ethnicity is reported, participants are predominantly white. The majority of participants are adolescents over the age of 11. The details of the included studies are presented in Table 2.

### Synthesis

The experience of having a parent with a life-limiting illness was grouped into four descriptive themes: the emotional impact of parental illness; the impact of caregiving; the need for openness; and managing the situation.

#### The emotional impact of parental illness

Having a parent with a life-limiting illness can have a profound emotional impact and cause psychological problems.^34–39,42,44,46–48,52,53^ Findings from included quantitative studies reveal that this cohort of children is more likely to display higher levels of anxiety and depression than control groups.^37,48,52,53^ This was supported by the qualitative data,^34–36,38,39,42,44,46,47^ where children reported that living with the constant threat of parental death causes an emotional burden, particularly having to live with uncertainty:

*‘I just wish I could control what’s going to happen – I just want to help her, but I can’t. . .and also, I’m really scared that some day, my dad’s cancer or my mum’s cancer returns, and then they’ll die – I’m scared that they’ll die. . .they mustn’t. . .I am really scared’* (p.231)^36^

Some participants expressed a feeling of responsibility for the parent’s illness, perhaps by having brought infection into the home or being helpless to provide comfort.^37,39,44,46,52,53^ Witnessing a parent’s illness could also evoke fears about the child’s own health and that of their siblings:

*‘I know there’s a chance you know of me and my sister both getting cancer when we’re older and I kind of think about that sometimes, whether I’d be able to cope with it in the same way that my mum did if I had that, or what kind of tests they’ll start doing on me and when they’ll start doing on me, and things like that’* (p.888)^42^

As an illness progresses, it can impact upon the availability of both parents, which can put a strain upon their relationship with their children and lead to children’s feelings of frustration and abandonment:

*‘Mom works full time, and she also has to deal with cancer, so she can’t do a lot of stuff, so that kind of, sometimes it can turn into like arguments, and sometimes you don’t have enough time with her to just like talk about like how her days were, like how our life is, and that can be hard sometimes. You lose, like touch’* (p.1061)^46^

#### The impact of caregiving

The included studies reveal that children of all ages regard themselves as a carer for their parent with a life-limiting illness, which can have both a negative and a positive impact.^34,35,38,39,41,46–48^ Having an ill parent causes additional burdensome responsibilities including household tasks, caring for younger siblings and providing personal care.^34,35,38,39,41,48^ There can be a disparity between the perceived amount of care provided, with children reporting higher levels of caregiving than parents.^34^ Findings from quantitative studies suggest that caregiving responsibilities and psychological problems in this cohort are connected, particularly in adolescents who are simultaneously striving for independence.^34,48^ When a parent has a life-limiting illness, the extent of household responsibilities were seen as being beyond those expected of their peers:

*‘What’s happening to my teenage years? They’ll be gone before you know it, and I’ll have spent them taking care of my little sisters, cleaning the house, and cooking. The rest of my friends are out having a good time’* (p.608)^39^

Time available for homework was reduced, impacting negatively upon school performance.^34,39,46^ The responsibility of being a young carer also affected participants’ time to engage in leisure activities or spend time with friends.^34,39,46^ The care provided to parents often extended beyond practical help into providing emotional support.^34,35,39,41^ Some participants described having a protective function, being well behaved and ensuring that a positive relationship was maintained:

*‘I’m less defiant, I guess. like I don’t want— like, there are a lot of people that don’t care about their parents, but like I don’t want to hurt my mom because I’m afraid that she could be gone anytime so I don’t want to mess up our relationship, so I’m very careful about that’* (p.1064)^46^

Caregiving was not always viewed negatively and some children appreciated the opportunity to care for their parent.^34,38,46,47^ Being a carer was associated with increased responsibility and trust, and participants described increased feelings of independence and resilience as a result of caregiving:

*‘it makes you more independent. You can’t rely on both parents, you rely on yourself and the other parent’* (p.26)^38^

#### The need for openness

It was apparent from included studies that children of all ages want to be aware of the life-limiting illness as soon as possible.^35,36,38,40–43,46,47,49,50,54^ However, children are not always kept updated: in one of the included quantitative studies, the majority of children expressed a desire for more information regarding their parent’s life-limiting illness.^40^ The importance of communication and information sharing was described throughout the studies, as openness enables children to feel prepared for the future and cope with the situation.^35,36,38,40–43,46,47,49,50,54^ Open communication within the family about the implications of the illness provides children with reassurance and helps to alleviate their emotional burden.^38,40,42,43,46,47,49,50^ Children were not always passive recipients of information, but exercised some control over the timing and content:

*‘I don’t want to understand like everything to do with it. It’s not something I really wanted to know. I don’t really want to know what the— like what cells or whatever it is that’s killing my Mum’* (p.417)^54^

Being uninformed about the illness can have a negative impact, affecting the relationship with parents and leading to distrust.^35,36,38,40,43^ Being shielded and protected by parents was often felt to be detrimental, creating feelings of isolation and marginalisation.^36,38,50^ Children are frequently more resilient and know more about the situation than given credit for:

*‘I mean, it’s no use hiding it because you figure out, like, if your parents don’t tell me, tell you and you, look at your dad and he’s stumbling around. You ask what happened? Then they will have to tell you, and then you cry even more. But if they tell you straight up, you like cry, and then you just get on with it’* (p.1065)^46^

#### Managing the situation

The final descriptive theme was the importance of developing coping strategies to help manage the situation.^36,39,41–44,46,47,49,51,54^ Children described a variety of strategies including having regular distractions away from home, talking about the illness and maximising time spent with their parents.^36,39,41–44,46,47,49,51,54^ For some children, an important coping strategy was to look for meaning and to acquire a deeper understanding of the situation.^36,39,42,46,47,49,51^ Others suggested that maintaining a positive attitude was vital when trying to manage the illness:

*‘I mean*, [the cancer is] *kind of frustrating, but I mean just adding you gotta think positively (. . .) This isn’t the end of the world. I mean, it’s sad, but you know, we’re going to get through it’* (p.1065)^46^

A strategy sometimes used by children was keeping the ‘ill world’ and the ‘well world’ separate.^36,39,41,47,51^ Maintaining a division between these worlds enables children to cope with parental life-limiting illness, whilst maintaining normalcy in aspects of their life. However keeping the two worlds apart can be problematic:

*‘When I wake up, it is a school day, but when I get back from school, it is basically like a workday because I am staying home, I am watching over the house, and I am taking care of everything there. It is kind of like I am a teenager and a grown-up. I go to school as a teenager. I get back, and I am not a teenager anymore. I am a grown-up, you know, and that alone, it could overload anybody’s head just thinking about it all’* (p.180)^51^

Having a confidant outside the family was also seen as desirable, for emotional support, information about the illness and advice about caregiving.^41–43^ However this support is unlikely to come from their peer group, teachers or healthcare professionals.^38,41–43^ Children did not feel included in the care provided to their family by palliative care teams.^42,43^ Seeking out other children facing parental death was considered beneficial and supportive:

*‘Support groups would definitely be nice. Just with, like, other people my age that are going through the same thing. So basically a reminder that I’m not the only one’* (p.19)^41^

## Discussion

The review has shown that the experience of living with a dying parent is emotionally demanding for children and can involve significant caregiving responsibilities. However these children are not passive, often developing strategies to cope with the situation and wanting to be as informed and involved as possible. This desire for agency represents a prevailing characteristic of this cohort. The concept of children having agency, whereby children have the right to assert their independence, make choices about their lives and contribute to their social world, is prevalent within current theories of childhood^55^ and is accepted as good practice internationally: both the United Nations (UN) Convention on the Rights of the Child^56^ and UK childcare legislation^57,58^ are founded upon the principles that children are active participants in their lives and have the right to be involved in matters which affect them. The concept of children maintaining their agency, despite parental life-limiting illness, is also consistent with other work in the field.^14,59^ In these studies, children living with parental life-limiting illness have been shown to be autonomous individuals, adopting strategies to manage a difficult situation and maintaining a sense of self and normality.

Childhood and adolescence is often a period of increasing independence and autonomy, with children trying to assert their agency, for example by developing political opinions that are not shared by their family.^60,61^ There can be tension and disagreement between children’s desire for increased freedom and self-reliance, with parents wanting to limit and contain their offspring’s developing agency.^62^ From the findings of the review, this tension appears to be intensified and further complicated by a parent’s life-limiting illness. Although the parent’s illness can be emotionally demanding, children in the included studies want to be informed about the situation and play an active role. This sense of responsibility towards the ill parent is one of the ways children attempt to create and maintain agency within their lives. This is often expressed by undertaking more domestic duties than would be expected of their age group, and there is a sense that children want to be emotionally caring too, for example by being well-behaved and maintaining a close relationship with their sick parent. Even younger children appear to consider themselves as having this duty of care towards their parent.

Although not necessarily associating themselves with the term ‘carer’, the children in this review provided both practical and emotional care to their parent with a life-limiting illness. Young carers are often not recognised as such^15^ and this appears to be the case with the children in this review. Many parents were unaware of the extent of caring responsibilities undertaken by their children or the sense of responsibility felt towards them.^34^ Healthcare professionals also do not appear to be identifying children living with parental life-limiting illness as carers and therefore not offering appropriate support. Not recognising young carers means that their needs are not addressed and that this vulnerable group can experience long-term problems.^63^ In order to address these poor outcomes, two pieces of legislation were introduced in England in 2014,^58,64^ placing a duty on professionals to identify and provide support to young carers. It is possible that the poor outcomes in people bereaved as children may be minimised if they are identified as young carers and their support needs addressed.

The review reveals that, from the perspectives of their children, parents affected by a life-limiting illness are unaware of the active contribution children make to the family and their social world. The agency of children living with parental life-limiting illness is often not recognised or acknowledged by the adults in their lives^59^ and this is supported by the review. Rather than seeing children as active participants, parents in the included studies tried to shield and protect them from the illness. Children were often not involved in conversations about the diagnosis, treatment and prognosis. Other studies have also shown that children are frequently excluded from conversations about their parent’s life-limiting illness.^35,65^ These attempts at protection may be well-meaning, however they inadvertently lead to ignorance, confusion and distrust.^66,67^ Associating children with death may challenge the common perception of childhood as a time of untainted innocence, and it is likely that parents want to preserve this innocence.^10,68^ It is argued that in Western societies, death has generally become a forbidden topic for discussion,^17,69,70^ and is especially considered a taboo subject for discussion with children.^71^ The attempt by parents in this review to shield their children from their life-limiting illness may therefore occur within the context of this cultural taboo. This taboo appears to be translated to healthcare professionals, who have been criticised for reinforcing the taboo around open discourse around death with children.^17^ Moreover, children in this review did not feel involved in the care provided to their families by palliative care teams.

Although themes were largely consistent across the review, two studies^44,45^ by the same authors contained an alternative perspective: the palliation stage of parental cancer is not necessarily the most stressful period during the illness and that family functioning may improve towards the end of life. A possible explanation for these confounding voices is that all of the participants in these studies were in receipt of counselling and may have developed coping strategies prior to the palliative stage. However this finding acts as a reminder of the diversity of experience of children facing the death of a parent and the variability of support offered to them.

The review displays a disconnect between children needing to be actively involved when a parent is dying, yet being excluded by adults under the guise of protection. On the one hand, children are trying to exert their agency and crave involvement when a parent is dying. On the other hand, both parents and healthcare professionals do not acknowledge this agency and aim to shield and exclude children. The result is that children are being denied their agency and their fundamental right to be involved in a hugely significant matter affecting them, the death of a parent.

## Implications for practice

Many healthcare professionals may be unfamiliar with the concept of children having agency. Considering children as active participants in their own lives when their parent has a life-limiting illness may be challenging, particularly in younger, pre-adolescent children. The profound emotional response experienced by parents can also occur in professionals, forcing them to question the widespread conception of childhood as a period of innocence.^68^ This may lead to a position of sympathy and over-protection, denying children their basic right to be involved in a major event affecting their life. Despite palliative care claiming to offer holistic care encompassing patients and their wider network,^72^ this holistic care does not appear to have been inclusive of the children in the included studies. This exclusion may be a result of the adult-centric view of the status of children and the limited opportunities for them to be recognised as being fully engaged in the complex emotional and social issues often accompanying parental life-limiting illness. Reflecting upon their personal stance on the issue of agency in children may be beneficial to professionals working with this cohort and may have an influence upon their practice.

A change in perception, acknowledging that all children living with parental life-limiting illness are potentially carers, may also lead to a more inclusive approach. Children are very likely to be providing care to their parents, and practitioners could act on this information by offering/signposting to appropriate support. The review has shown that even children under 10 are involved in caregiving. Every local authority in the UK has an organisation providing support to young carers,^13^ so suggesting a referral on diagnosis of a parental life-limiting illness may be beneficial. Flexible approaches with families, seeking out ‘invisible’ children who may not come into direct contact with the clinical team, but are nonetheless directly impacted by the parent’s illness, could be considered by professionals. School-age children in the review reported that professionals tended to make home visits during school hours, so they never had any direct contact with them. A simple change in working hours may be more inclusive of children and is an approach recommended elsewhere.^73^ Not recognising or addressing the contribution that children make as young carers of parents with life-limiting illnesses may have a significant impact on how they manage the situation and further contribute to their long-term negative outcomes.

Conceptualising children as carers may also have an impact upon the extent to which children are informed about the prognosis. From the findings of the review, children want openness about the parental life-limiting illness from the onset. It is unlikely that adult carers would be excluded from information about prognosis, so perceiving children as carers may also ensure their involvement. Norway and Sweden have taken a progressive approach by enacting legislation placing a duty on healthcare professionals to actively involve children when a parent is dying.^74–76^ Children of ill parents are referred to as ‘next-of-kin’ or ‘relatives’ by professionals in these countries, reflecting how they are perceived.^77–79^ This is a recognition of children’s agency and their fundamental right to be involved in decisions affecting them, as enshrined within the UN Convention on the Rights of the Child.^56^ The findings of this review support the pioneering stance taken by Norway and Sweden and demonstrate children’s desire to be informed and involved when a parent is dying. Professionals working with parents with a life-limiting illness have a responsibility to talk about the importance of communicating with children about the illness. Even younger, pre-adolescent children will have an awareness that something is wrong and have a right to age-appropriate information. Guidance and support about how to initiate such conversations could be offered, for example parents can be directed towards resources giving advice on how to have an open dialogue around the prognosis.^80,81^ The potential detrimental impact of not being open about the prognosis with children should be stressed to parents.

## Strengths and limitations

This is the first review to focus exclusively on the direct experience of children facing the death of a parent, enabling the voices of this vulnerable cohort to be expressed. The use of integrative review methodology enabled the inclusion of both qualitative and quantitative data. However the review is based upon the findings of studies that are of variable quality. The voices of included children are predominantly those of adolescents living with parental cancer in Western countries and where specified, the majority of participants are from a White background. The findings are therefore not necessarily transferable to all children living with parental life-limiting illness. All of the included papers are in the English language. Due to the large number of papers identified for title and abstract review, it is possible that relevant articles may have been omitted, although having two authors undertake the screening ensured this risk was minimised.

## Conclusion

The review has shown that children facing parental life-limiting illness strive to maintain their agency. Despite the emotional impact and additional caregiving responsibilities associated with having a seriously ill parent, children continue to make independent choices and adopt strategies to manage their changed social world. Children maintaining agency in the face of parental life-limiting illness is a useful conceptualisation of their experience, and may be beneficial to healthcare professionals. There is international recognition that children have a fundamental right to be involved in matters affecting them, and a changed perspective on children’s rights by professionals may facilitate this. Regarding children as active participants when a parent is dying, even considering them to have the status of a carer, might provide professionals with a novel perspective on children’s role and position in families. Parents will be inclined towards overprotection and shielding, and professionals are able to use the evidence within this review to show how this is unhelpful, contrary to children’s wishes and may lead to long-term emotional problems. The review has reinforced an unequivocal message that healthcare professionals can utilise in their interactions with dying parents: children want to know what is happening and want to play an active role.
